# Predicting overactive bladder from inflammatory markers: A machine learning approach using NHANES 2005–2020

**DOI:** 10.17305/bb.2025.12335

**Published:** 2025-05-14

**Authors:** Haoxun Zhang, Guoling Zhang, Chunyang Wang

**Affiliations:** 1 Department of Urology, The First Affiliated Hospital of Harbin Medical University, Harbin, China

**Keywords:** Overactive bladder, OAB, inflammatory biomarkers, machine learning, National Health and Nutrition Examination Survey, NHANES, predictive modeling

## Abstract

Overactive bladder (OAB), a prevalent condition characterized by urgency and nocturia, imposes significant burdens on both quality of life and healthcare systems. Emerging evidence implicates systemic inflammation in OAB pathogenesis; however, the role of complete blood count (CBC)-derived inflammatory biomarkers remains underexplored. This cross-sectional study analyzed data from 35,394 participants in the National Health and Nutrition Examination Survey (NHANES, 2005–2020) to evaluate associations between CBC-derived biomarkers—such as the Systemic Immune-Inflammation Index (SII), Systemic Inflammation Response Index (SIRI), and Neutrophil-to-Lymphocyte Ratio (NLR)—and OAB (defined by an OAB Symptom Score ≥3). Multivariable logistic regression, threshold analysis, and machine learning models (Random Forest [RF], Extreme Gradient Boosting) were employed, adjusting for sociodemographic, lifestyle, and clinical covariates. Elevated levels of SII, SIRI, NLR, Monocyte-to-Lymphocyte Ratio (MLR), and Neutrophil-MLR (NMLR) were significantly associated with increased OAB risk (all *P* < 0.05), with adjusted odds ratios for the highest quartiles ranging from 1.21 (SII; 95% CI: 1.10–1.34) to 1.31 (NMLR; 1.19–1.44). Nonlinear associations were observed, with inflection points (e.g., NLR ═ 1.071, MLR ═ 0.174) marking abrupt increases in risk. RF models showed strong predictive performance (area under the curve ═ 0.89 for training; 0.76 for testing), identifying SII and SIRI as key predictors. Subgroup analyses demonstrated consistent associations across most demographic groups, with the exception of hyperlipidemia, which modified the effects of SIRI, NLR, and NMLR. These findings highlight the role of systemic inflammation in OAB and suggest that CBC-derived biomarkers could serve as cost-effective tools for risk stratification. The integration of epidemiological analysis and machine learning enhances our understanding of OAB’s inflammatory underpinnings, although longitudinal studies are needed to establish causal relationships and therapeutic implications.

## Introduction

Overactive bladder (OAB) is defined by the International Continence Society as urinary urgency, usually accompanied by frequency and nocturia, with or without urgency urinary incontinence, in the absence of urinary tract infection or other identifiable pathology [1]. Although patients typically do not present with obvious clinical abnormalities (such as urinary tract infections), common symptoms include increased daytime frequency and nocturia [2]. An epidemiological survey in China reported an overall OAB prevalence of approximately 6.0% [3]. In the United States, the prevalence among adult males and females is 16% and 16.9%, respectively, with rates increasing with age [4]. Despite its prevalence, OAB is often underdiagnosed in both men and women, with only a minority of affected individuals seeking treatment. Notably, OAB significantly diminishes quality of life, interferes with daily activities, and can lead to depression or anxiety [5]. Current treatment options have notable limitations and are frequently associated with adverse effects. Additionally, the aging population contributes to a growing OAB burden, underscoring its importance as a healthcare challenge [6]. In the U.S., OAB imposes a substantial annual economic burden, with healthcare costs for patients exceeding those of non-OAB individuals by more than 2.5 times—making it a pressing public health concern [7]. Nevertheless, the risk factors and underlying pathological mechanisms of OAB remain poorly understood. Emerging research suggests that immune-inflammatory responses may play a key role in OAB pathogenesis [8]. Studies have identified significantly elevated levels of inflammatory markers—including C-reactive protein, prostaglandins, adipokines, nerve growth factor, and brain-derived neurotrophic factor—in the serum and urine of OAB patients, suggesting that heightened inflammation may contribute to the condition’s clinical features [9–12]. Inflammation may lead to peripheral nerve sensitization, which could trigger hallmark symptoms, such as increased urinary frequency and urgency. Clinically, white blood cell counts and their differential subtypes serve as accessible surrogate markers for evaluating systemic inflammation [13]. Neutrophils—the most abundant white blood cells—initiate innate immune responses and are involved in both acute injury repair and chronic inflammation. Monocytes contribute to pathogen clearance and the removal of damaged cells during inflammation [14, 15]. Lymphocytes, which govern both cellular and humoral immune responses, regulate the overall inflammatory environment [16]. The dynamic interplay among these immune cells is essential for immune surveillance, defense, and homeostasis; disruptions in this balance may contribute to disease development. Research into inflammation-related biomarkers has gained momentum, particularly with the rise of novel indices derived from complete blood count (CBC) data. Biomarkers, such as the Systemic Immune-Inflammation Index (SII), Systemic Inflammation Response Index (SIRI), Neutrophil-to-High-Density Lipoprotein Ratio (NHR), Neutrophil-to-Lymphocyte Ratio (NLR), Monocyte-to-Lymphocyte Ratio (MLR), and Neutrophil-MLR (NMLR) offer insights into both localized immune activity and overall systemic inflammation [17–19]. However, the relationship between these CBC-derived inflammatory markers and OAB remains insufficiently explored. Therefore, this study leverages publicly available data from the National Health and Nutrition Examination Survey (NHANES) to examine the association between systemic inflammation—as measured by CBC-derived biomarkers—and OAB in a cross-sectional analysis. The goal is to better understand the potential connection between inflammation and OAB, ultimately informing improved clinical prevention and treatment strategies.

## Materials and methods

### Study population

**Figure 1.**
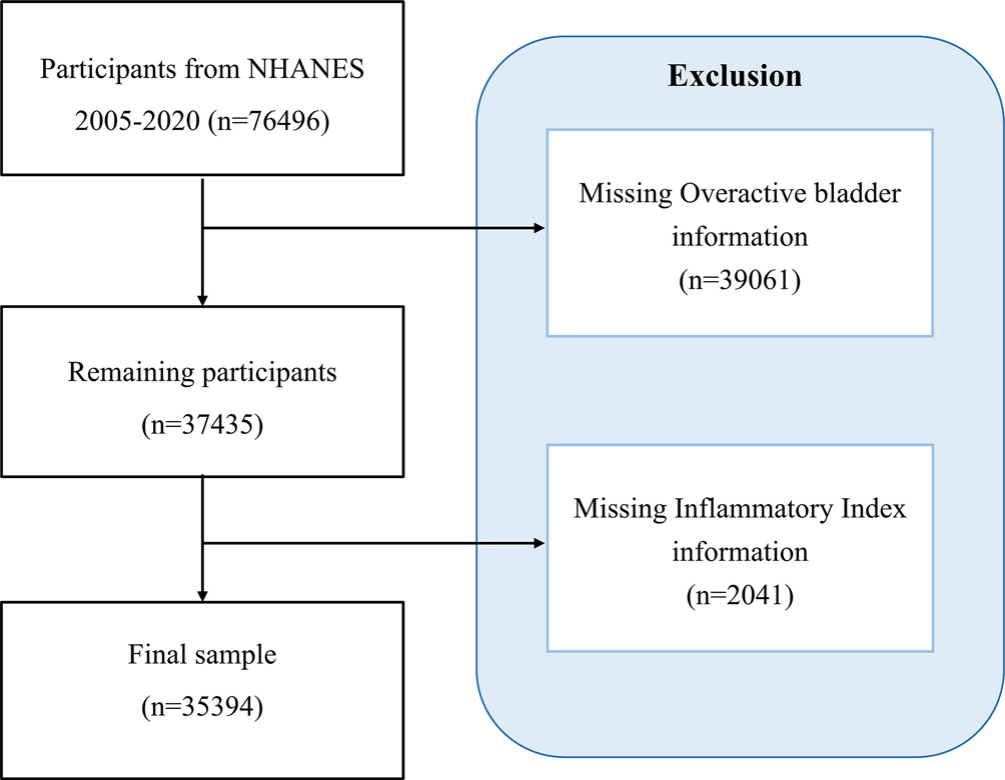
**Flowchart of participant selection from the NHANES 2005–2020 dataset.** NHANES: National Health and Nutrition Examination Survey.

The data used in this study were obtained from the NHANES, https://wwwn.cdc.gov/nchs/nhanes/Default.aspx, a nationally representative, open-access survey that collects dietary, demographic, physical, and medical information to assess the health status of the U.S. civilian population. Between 2005 and 2020, a total of 76,496 participants were initially screened across eight survey cycles: 2005–2006, 2007–2008, 2009–2010, 2011–2012, 2013–2014, 2015–2016, 2017–2018, and 2019–2020. Participants with incomplete data on OAB syndrome (*n* ═ 39,061) and those lacking information on inflammatory markers (*n* ═ 2041) were excluded, resulting in a final sample of 35,394 individuals for further analysis. Figure 1 presents a detailed flowchart of the participant selection process. As this study involved secondary analysis of publicly available, anonymized cross-sectional data, additional institutional review board approval was not required.

### Assessment of CBC-derived inflammatory biomarkers

CBCs were performed using a Coulter^®^ DxH 800 analyzer, with results expressed as ×10^3^ cells/µL. Based on these data, six systemic inflammation markers were calculated: the SII, SIRI, NHR, NLR, MLR, and NMLR. The formulas are as follows:

SII ═ (Platelet count × Neutrophil count)/Lymphocyte count

SIRI ═ (Neutrophil count × Monocyte count)/Lymphocyte count

NHR ═ Neutrophil count/High-density Lipoprotein cholesterol

NLR ═ Neutrophil count/Lymphocyte count

MLR ═ Monocyte count/Lymphocyte count

NMLR ═ (Neutrophil count + Monocyte count)/Lymphocyte count.

### Definition of OAB

Data on OAB were collected using the NHANES Kidney Disease-Urology Questionnaire, which included questions about urgency urinary incontinence and nocturia. The severity of urgency incontinence was assessed through two items: (1) In the past 12 months, have you leaked or lost control of your urine due to a sudden urge or pressure to urinate and couldn’t get to the toilet fast enough? (2) “How often does this happen?” The severity of nocturia was evaluated based on another question: “In the last 30 days, from going to bed at night to getting up in the morning, how many times per night do you typically wake up to urinate?” Symptom severity was quantified using the validated OAB Symptom Score (OABSS) developed by Blaivas et al. [20, 21]. Scoring criteria (detailed in Table S1) assigned points based on the frequency of urgency incontinence episodes and nocturia. Each subject’s OABSS in NHANES was calculated by summing their urgency incontinence and nocturia scores. A cumulative score of ≥3 was used as the diagnostic threshold for OAB.

### Covariates

The study included the following covariates: gender (male/female), age (in years), race/ethnicity (Mexican American, Other Hispanic, Non-Hispanic White, Non-Hispanic Black, Other Race), education level (less than 9th grade, 9–11th grade, high school graduate, some college or associate degree, college graduate or above), marital status (married or living with a partner, divorced/separated/widowed, never married), poverty-income ratio (PIR; <1.3, 1.3–1.85, 1.85–3.5, >3.5), body mass index (BMI: <25, 25–30, >30 kg/m^2^), alcohol use history, smoking history, and diagnoses of diabetes, hypertension, hyperlipidemia, and coronary heart disease (CHD; all recorded as Yes/No).

### Ethical statement

The NHANES protocol was approved by the Research Ethics Review Board of the CDC’s National Center for Health Statistics, and all participants provided written informed consent. This study used anonymized secondary data and did not involve direct interaction with human subjects.

### Statistical analysis

CBC-derived inflammatory markers were categorized into quartiles, with the lowest quartile serving as the reference group. To meet the normality assumptions required for statistical analysis, a log_10_ transformation was applied to the SII markers, enhancing interpretability. Continuous variables were reported as mean ± standard deviation, while categorical variables were presented as proportions. Group comparisons were conducted using chi-square tests for categorical variables and *t*-tests for continuous variables. A total of 35,394 participants were randomly split into training (*n* ═ 24,775) and testing (*n* ═ 10,619) cohorts using a 7:3 ratio. Variable selection was carried out using the extended Boruta algorithm and Least Absolute Shrinkage and Selection Operator (LASSO) regression. The common variables selected—gender, age, race, education, marital status, PIR, BMI, smoking status, hypertension, diabetes, SII, and SIRI—were used to construct predictive models. The random forest (RF) prediction model was developed using the randomForest package in R, with hyperparameters specified as follows: ntree ═ 500 (number of trees), mtry ═ sqrt(p) (number of variables randomly sampled at each split, where p is the total number of predictors), and nodesize ═ 1 (minimum terminal node size for classification). Model performance was evaluated using 10-fold cross-validation stratified by outcome prevalence. Missing covariates were imputed using the Joint Modeling Multiple Imputation (JOMO) method with 10 imputations. Convergence was assessed using 500 burn-in iterations and 100 iterations between imputations [22]. The imputation model included complete covariates (e.g., age, gender, and race) and SDMVPSU. All exposure variables (CBC-derived biomarkers) and the outcome variable (OAB diagnosis) were included in the imputation under the missing-at-random assumption. Participants with missing exposure or outcome data were retained during imputation but excluded from the final complete-case analysis. Sensitivity analyses under missing-not-at-random (MNAR) assumptions were performed by systematically varying key imputed variables (e.g., CBC biomarkers) using delta adjustments (±0.5 SD). The results remained robust, with <5% change in the primary association effect estimates (Table S2). Statistical significance was defined as *P* < 0.05. All analyses were conducted using R software (version 4.3.1) and EmpowerStats (version 4.0).

## Results

### Participant characteristics

Table 1 summarizes the comparison of CBC-derived inflammatory biomarker levels and key demographic variables between subjects with and without OAB. Of the 35,394 participants included, 27,864 (78.73%) did not have OAB, while 7530 (21.27%) were diagnosed with the condition. Females accounted for 51% of the study population, and the weighted mean age was 49.94 years (SD ═ 17.76). Table 2 indicates that clinical characteristics—including obesity, alcohol consumption, smoking, and comorbidities, such as diabetes, hypertension, hyperlipidemia, and CHD—were significantly associated with the prevalence of OAB. All CBC-derived biomarkers—SII, SIRI, NHR, NLR, MLR, and NMLR—were significantly elevated in OAB patients compared to controls (*P* < 0.001). For example, the mean SII was 597.25 in the OAB group vs 540.35 in the control group, while the mean SIRI was 1.43 compared to 1.25 in non-OAB participants.

**Table 1:** Demographic characteristics of participants from NHANES 2005–2020

**Variables**	**Overall**	**OAB**		***P* value**
	**(*n* ═ 35,394)**	**NO (*n* ═ 27,864)**	**YES (*n* ═ 7530)	
Gender, *n*(%)				0.261
Male	17,422 (49%)	13,683 (49%)	3739 (50%)	
Female	17,972 (51%)	14,181 (51%)	3791 (50%)	
Age, Mean + SD	49.94 ± (17.76)	49.91 ± (17.72)	50.05 ± (17.93)	0.747
Age strata, *n*(%)				0.773
20–40	11,694 (33%)	9212 (33%)	2482 (33%)	
40–60	11,631 (33%)	9177 (33%)	2454 (33%)	
≥60	12,069 (34%)	9475 (34%)	2594 (35%)	
Race, *n*(%)				0.101
Mexican American	5452 (15%)	4288 (15%)	1164 (15%)	
Other Hispanic	3459 (9.8%)	2761 (9.9%)	698 (9.2%)	
Non-Hispanic White	15,193 (43%)	11,932 (43%)	3261 (44%)	
Non-Hispanic Black	7549 (21%)	5994 (21%)	1555 (20%)	
Other race	3741 (11%)	2889 (10%)	852 (12%)	
Education level, *n*(%)				0.214
Less than 9th grade	3519 (9.5%)	2794 (9.5%)	725 (9.4%)	
9–11th grade	4859 (14%)	3834 (14%)	1025 (14%)	
High school graduate	8188 (23%)	6434 (23%)	1754 (22%)	
Some college or associates degree	10,630 (30%)	8410 (30%)	2220 (29%)	
College graduate or above	8198 (24%)	6392 (23%)	1806 (25%)	
Marital status, *n*(%)				0.394
Married/Living with a partner	21,156 (60%)	16,594 (60%)	4562 (61%)	
Divorced/Separated/Widowed	9284 (26%)	7367 (26%)	1917 (25%)	
Never married	4954 (14%)	3903 (14%)	1051 (14%)	
Poverty-income ratio, *n*(%)				0.220
<1.3	10,343 (29%)	8225 (29%)	2118 (28%)	
1.3–1.85	4754 (13%)	3746 (13%)	1008 (14%)	
1.85–3.5	9513 (27%)	7524 (27%)	1989 (26%)	
≥3.5	10,784 (32%)	8369 (31%)	2415 (33%)	

NHANES: National Health and Nutrition Examination Survey; OAB: Overactive bladder.

**Table 2:** Clinical characteristics of participants from NHANES 2005–2020

**Variables**	**Overall**	**OAB**		***P* value**
	**(*n* ═ 35,394)**	**NO (*n* ═ 27,864)**	**YES (*n* ═ 7530)**	
BMI, *n(%)*				<0.001
<25	9836 (29%)	8362 (31%)	1474 (20%)	
25–30	11,718 (33%)	9520 (34%)	2198 (30%)	
≥30	13,840 (38%)	9982 (36%)	3858 (50%)	
Alcohol use, *n(%)*				<0.001
Yes	26,706 (81%)	21,371 (82%)	5335 (75%)	
No	8688 (19%)	6493 (18%)	2195 (25%)	
Smoking, *n(%)*				<0.001
Yes	15,864 (45%)	12,080 (44%)	3784 (51%)	
No	19,530 (55%)	15,784 (56%)	3746 (49%)	
Diabetes, *n(%)*				<0.001
Yes	4869 (10%)	2903 (8.1%)	1966 (22%)	
No	30,525 (90%)	24,961 (92%)	5564 (78%)	
Hypertension, *n(%)*				<0.001
Yes	12,928 (32%)	8674 (28%)	4254 (52%)	
No	22,466 (68%)	19,190 (72%)	3276 (48%)	
Hyperlipidemia, *n(%)*				<0.001
Yes	13,289 (36%)	9636 (34%)	3653 (48%)	
No	22,105 (64%)	18,228 (66%)	3877 (52%)	
Coronary heart disease, *n(%)*				<0.001
Yes	1488 (3.6%)	914 (2.9%)	574 (7.4%)	
No	33,906 (96%)	26,950 (97%)	6956 (93%)	
SII, Mean + SD	549.70 ± (336.72 )	540.35 ± (314.28)	597.25 ± (430.10)	<0.001
SIRI, Mean + SD	1.28 ± (0.89)	1.25 ± (0.84)	1.43 ± (1.07)	<0.001
NHR, Mean + SD	3.46 ± (1.92)	3.43 ± (1.86)	3.62 ± (2.22)	<0.001
NLR, Mean + SD	2.2 1 ± (1.16)	2.17 ± (1.11)	2.40 ± (1.37)	<0.001
MLR, Mean + SD	0.29 ± (0.13)	0.28 ± (0.12)	0.31 ± (0.14)	<0.001
NMLR, Mean + SD	2.50 ± (1.24)	2.46 ± (1.19)	2.71 ± (1.47)	<0.001

NHANES: National Health and Nutrition Examination Survey; OAB: Overactive bladder; SII: Systemic Immune-Inflammation Index; SIRI: Systemic Inflammation Response Index; NHR: Neutrophil-to-High-Density Lipoprotein Ratio; NLR: Neutrophil-to-Lymphocyte Ratio; MLR: Monocyte-to-Lymphocyte Ratio; NMLR: Neutrophil-MLR.

### Multivariate logistic regression models

Four groups were established based on CBC-derived biomarkers, and logistic regression analyses revealed a positive correlation between increasing biomarker quartiles and the incidence of OAB. In Model 1, a dose-response relationship was observed, which persisted after adjusting for age, race, and gender. In Model 2, higher levels of CBC-derived biomarkers remained significantly associated with an increased prevalence of OAB. In Model 3—adjusted for all covariates—significant associations were maintained for all biomarkers except NHR. The odds ratios comparing the highest to the lowest quartile were as follows: log10 (SII) (1.21 [1.10–1.34]), SIRI (1.28 [1.16–1.41]), NHR (1.11 [0.98–1.25]), NLR (1.30 [1.18–1.44]), MLR (1.27 [1.15–1.40]), and NMLR (1.31 [1.19–1.44]) (Table 3).

**Table 3:** Association between CBC-derived inflammatory biomarkers and OAB

	**Model 1** **OR (95% CI)**	**Model 2** **OR (95% CI)**	**Model 3** **OR (95% CI)**
*SII*			
Continuous	1.56 (1.40, 1.73)	1.56 (1.41, 1.73)	1.33 (1.19, 1.48)
Q1	Reference	Reference	Reference
Q2	0.88 (0.80, 0.98)	0.88 (0.80, 0.98)	0.88 (0.80, 0.98)
Q3	1.02 (0.92, 1.14)	1.02 (0.92, 1.14)	0.96 (0.86, 1.06)
Q4	1.39 (1.26, 1.54)	1.40 (1.26, 1.54)	1.21 (1.10, 1.34)
*P* for trend	<0.001	<0.001	<0.001
*SIRI*			
Continuous	1.23 (1.19, 1.26)	1.23 (1.19, 1.26)	1.14 (1.10, 1.17)
Q1	Reference	Reference	Reference
Q2	0.93 (0.84, 1.04)	0.93 (0.84, 1.04)	0.90 (0.81, 1.01)
Q3	1.09 (0.97, 1.22)	1.09 (0.97, 1.22)	0.97 (0.86, 1.09)
Q4	1.59 (1.45, 1.75)	1.60 (1.46, 1.76)	1.28 (1.16, 1.41)
*P* for trend	<0.001	<0.001	<0.001
*NHR*			
Continuous	1.05 (1.03, 1.07)	1.05 (1.04, 1.07)	1.03 (1.01, 1.06)
Q1	Reference	Reference	Reference
Q2	1.00 (0.90, 1.11)	1.01 (0.90, 1.13)	1.00 (0.89, 1.11)
Q3	1.08 (0.97, 1.21)	1.10 (0.98, 1.23)	1.07 (0.95, 1.21)
Q4	1.21 (1.10, 1.33)	1.24 (1.12, 1.37)	1.11 (0.98, 1.25)
*P* for trend	<0.001	<0.001	0.067
*NLR*			
Continuous	1.16 (1.12, 1.19)	1.16 (1.12, 1.19)	1.10 (1.07, 1.13)
Q1	Reference	Reference	Reference
Q2	0.92 (0.82, 1.03)	0.92 (0.82, 1.03)	0.90 (0.80, 1.02)
Q3	1.08 (0.98, 1.20)	1.08 (0.98, 1.20)	0.98 (0.89, 1.09)
Q4	1.56 (1.42, 1.72)	1.56 (1.42, 1.72)	1.30 (1.18, 1.44)
*P* for trend	<0.001	<0.001	<0.001
*MLR*			
Continuous	3.52 (2.71, 4.58)	3.54 (2.73, 4.59)	2.38 (1.83, 3.10)
Q1	Reference	Reference	Reference
Q2	0.90 (0.82, 1.00)	0.91 (0.82, 1.00)	0.92 (0.82, 1.02)
Q3	0.97 (0.87, 1.07)	0.97 (0.88, 1.07)	0.96 (0.86, 1.08)
Q4	1.44 (1.31, 1.59)	1.45 (1.32, 1.59)	1.27 (1.15, 1.40)
*P* for trend	<0.001	<0.001	<0.001
*NMLR*			
Continuous	1.15 (1.12, 1.18)	1.15 (1.12, 1.18)	1.09 (1.07, 1.12)
Q1	Reference	Reference	Reference
Q2	0.88 (0.79, 0.99)	0.89 (0.79, 0.99)	0.87 (0.77, 0.98)
Q3	1.02 (0.92, 1.12)	1.02 (0.92, 1.12)	0.93 (0.84, 1.03)
Q4	1.56 (1.43, 1.71)	1.57 (1.43, 1.72)	1.31 (1.19, 1.44)
*P* for trend	<0.001	<0.001	<0.001

CBC: Complete blood count; OAB: Overactive bladder; SII: Systemic Immune-Inflammation Index; SIRI: Systemic Inflammation Response Index; NHR: Neutrophil-to-High-Density Lipoprotein Ratio; NLR: Neutrophil-to-Lymphocyte Ratio; MLR: Monocyte-to-Lymphocyte Ratio; NMLR: Neutrophil-MLR.

### Smooth curve fitting (SCF) and threshold effect analysis

**Figure 2.**
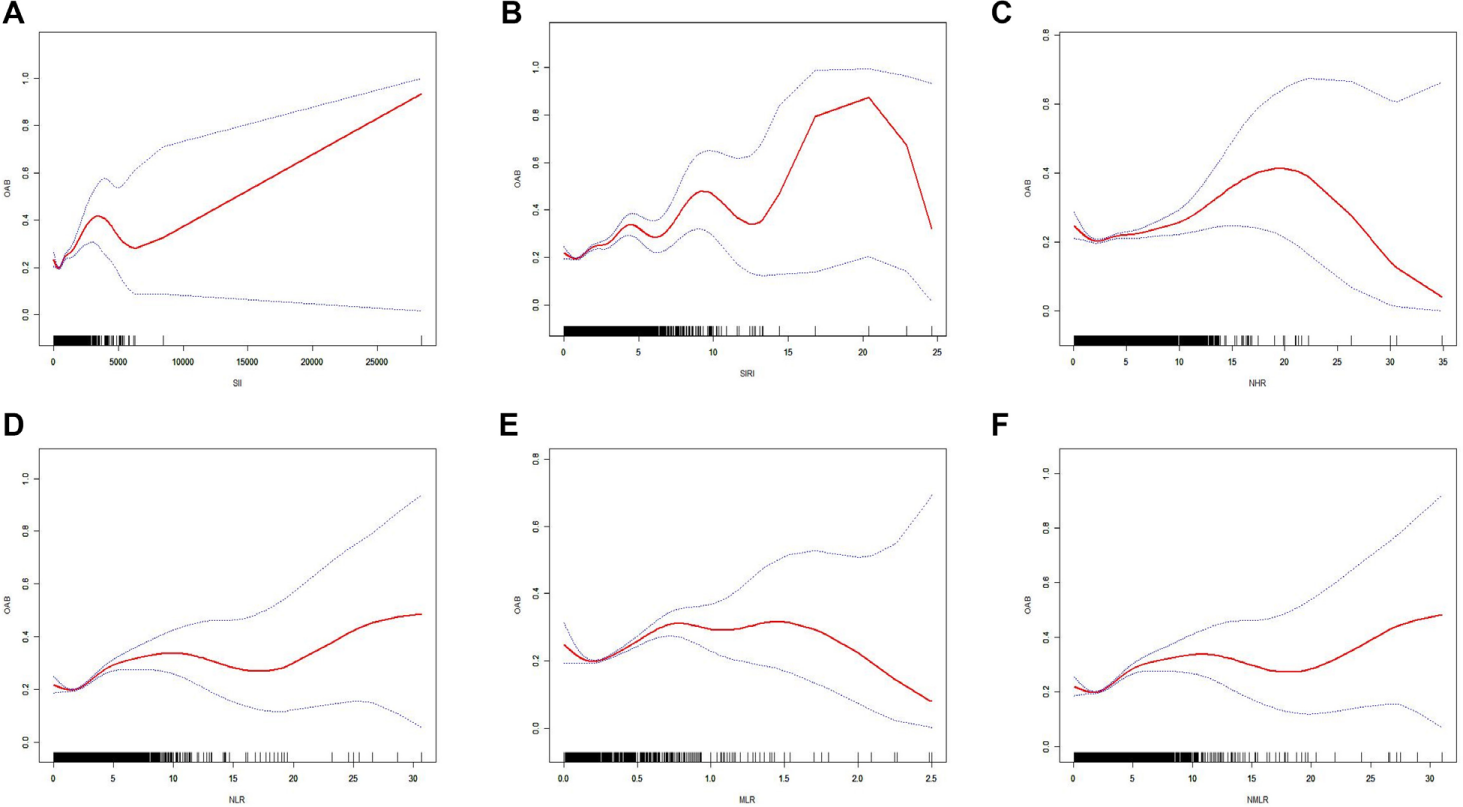
**The nonlinear relationship between inflammatory index and OAB.** The solid red line indicates a smooth curve fit between the variables. The blue band indicates the 95% confidence interval of the fit. (A) SII; (B) SIRI; (C) NHR; (D) NLR; (E) MLR; (F) NMLR. OAB: Overactive bladder; SII: Systemic Immune-Inflammation Index; SIRI: Systemic Inflammation Response Index; NHR: Neutrophil-to-High-Density Lipoprotein Ratio; NLR: Neutrophil-to-Lymphocyte Ratio; MLR: Monocyte-to-Lymphocyte Ratio; NMLR: Neutrophil-MLR.

To further investigate the relationship between CBC-derived biomarkers and OAB, we conducted an SCF analysis using a generalized additive model based on Model 3. As shown in Figure 2, the SCF analysis revealed significant nonlinear associations between all CBC-derived biomarkers and the incidence of OAB (all *P* < 0.05). To quantify these nonlinear relationships, we performed a threshold effect analysis, which identified distinct inflection points for each biomarker: 0.272 for log_10_ (SII), 0.467 for SIRI, 1.436 for NHR, 1.071 for NLR, 0.174 for MLR, and 1.114 for NMLR (Table 4). The log-likelihood ratio tests for all threshold models were statistically significant (*P* < 0.05), supporting the robustness of these nonlinear associations.

**Table 4:** Threshold effect analysis of CBC-derived inflammatory biomarkers on OAB using a two-piecewise logistic regression model in the NHANES 2005–2020

**Threshold effect analysis**	**OAB** **OR (95% CI) *P* value**
*SII*	
Inflection point (K)	0.272
<K slope	0.232 (0.093, 0.578) 0.0017
>K slope	1.444 (1.333, 1.564) < 0.0001
Log-likelihood ratio test	<0.001
*SIRI*	
Inflection point (K)	0.467
<K slope	0.540 (0.273, 1.070) 0.0775
>K slope	1.164 (1.130, 1.199) < 0.001
Log-likelihood ratio test	0.031
*NHR*	
Inflection point (K)	1.436
<K slope	0.613 (0.478, 0.787) 0.0001
>K slope	1.034 (1.018, 1.050) < 0.001
Log-likelihood ratio test	<0.001
*NLR*	
Inflection point (K)	1.071
<K slope	0.644 (0.462, 0.900) 0.0099
>K slope	1.117 (1.092, 1.142) < 0.001
Log-likelihood ratio test	0.002
*MLR*	
Inflection point (K)	0.174
<K slope	0.061 (0.008, 0.441) 0.0056
>K slope	2.542 (2.037, 3.172) < 0.001
Log-likelihood ratio test	<0.001
*NMLR*	
Inflection point (K)	1.114
<K slope	0.539 (0.346, 0.839) 0.0063
>K slope	1.109 (1.086, 1.132) < 0.001
Log-likelihood ratio test	0.002

CBC: Complete blood count; OAB: Overactive bladder; SII: Systemic Immune-Inflammation Index; SIRI: Systemic Inflammation Response Index; NHR: Neutrophil-to-High-Density Lipoprotein Ratio; NLR: Neutrophil-to-Lymphocyte Ratio; MLR: Monocyte-to-Lymphocyte Ratio; NMLR: Neutrophil-MLR.

**Figure 3.**
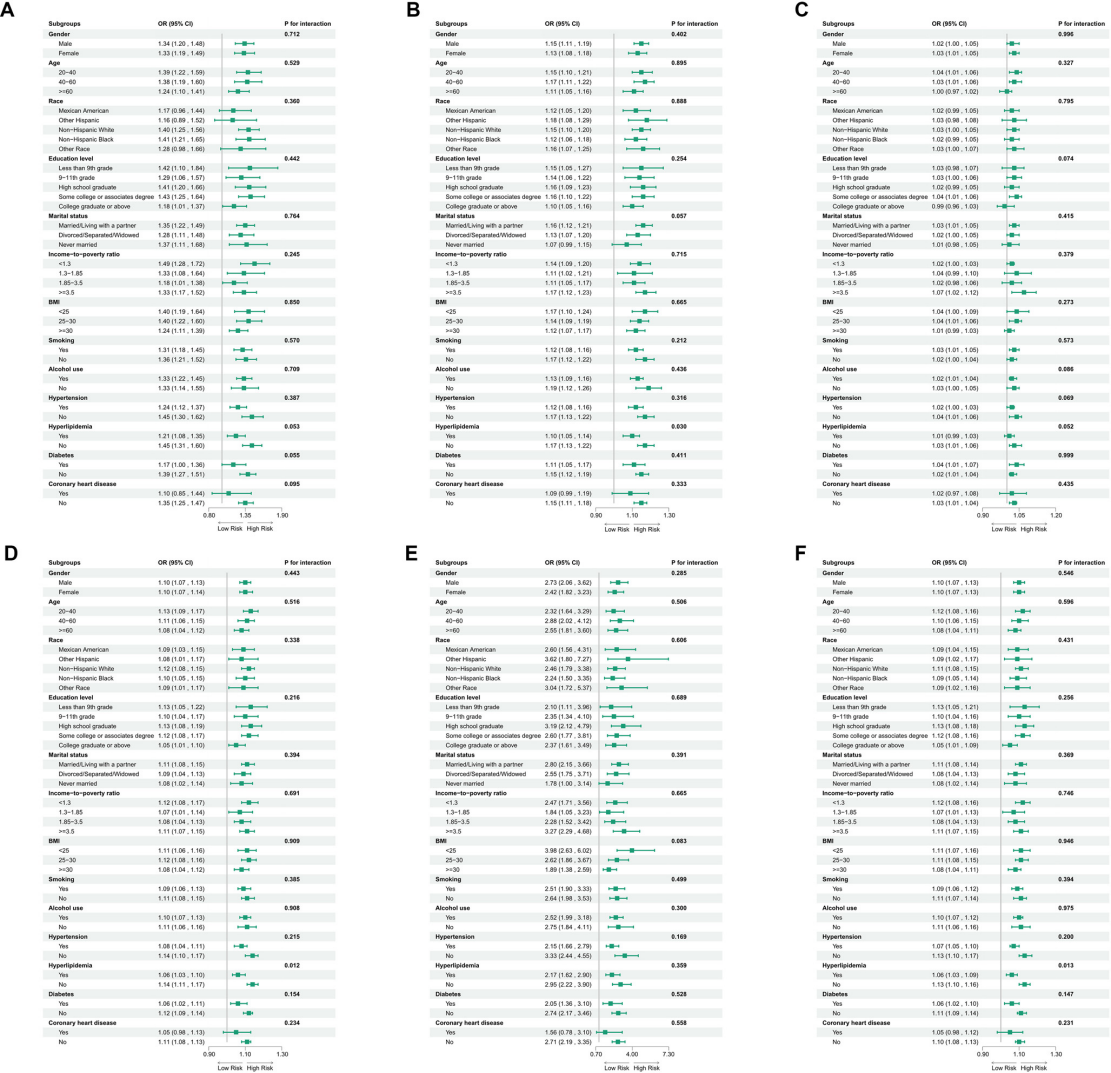
**Subgroup analysis of the association between CBC-derived inflammatory biomarkers and OAB risk across various stratification factors, including gender, age, race, education, marital status, PIR, BMI, smoking status, alcohol use, and comorbidities.** (A) SII; (B) SIRI; (C) NHR; (D) NLR; (E) MLR; (F) NMLR. CBC: Complete blood count; OAB: Overactive bladder; SII: Systemic Immune-Inflammation Index; SIRI: Systemic Inflammation Response Index; NHR: Neutrophil-to-High-Density Lipoprotein Ratio; NLR: Neutrophil-to-Lymphocyte Ratio; MLR: Monocyte-to-Lymphocyte Ratio; NMLR: Neutrophil-MLR; BMI: Body mass index; PIR: Poverty-income ratio.

**Figure 4.**
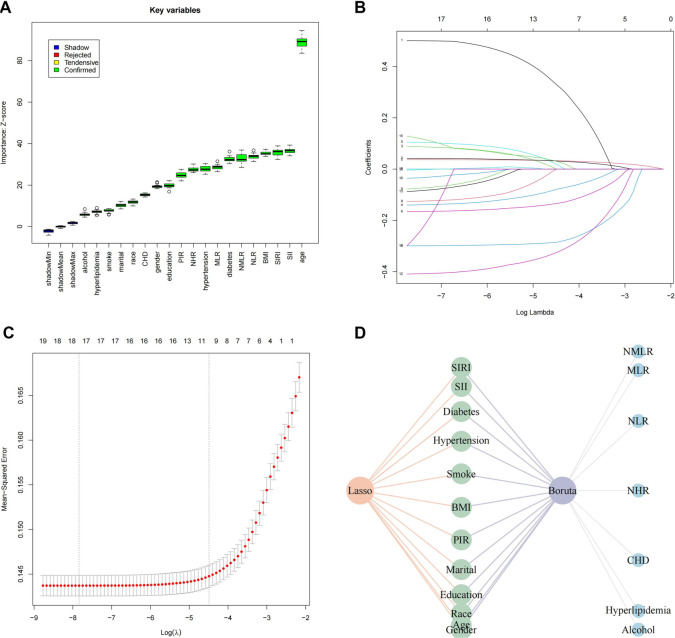
**Predictor screening results.** (A) Boruta; (B) Factor screening based on the LASSO regression model, with the left dashed line indicating the best lambda value for the evaluation metrics (lambda. min) and the right dashed line indicating the lambda value for the model where the evaluation metrics are in the range of the best value by one standard error (lambda. 1se ═ 0.01121794); (C) LASSO regression model screening variable trajectories; (D) Common predictors between Boruta and LASSO. LASSO: Least Absolute Shrinkage and Selection Operator; CHD: Coronary heart disease; SII: Systemic Immune-Inflammation Index; SIRI: Systemic Inflammation Response Index; NHR: Neutrophil-to-High-Density Lipoprotein Ratio; NLR: Neutrophil-to-Lymphocyte Ratio; MLR: Monocyte-to-Lymphocyte Ratio; NMLR: Neutrophil-MLR; BMI: Body mass index; PIR: Poverty-income ratio.

**Figure 5.**
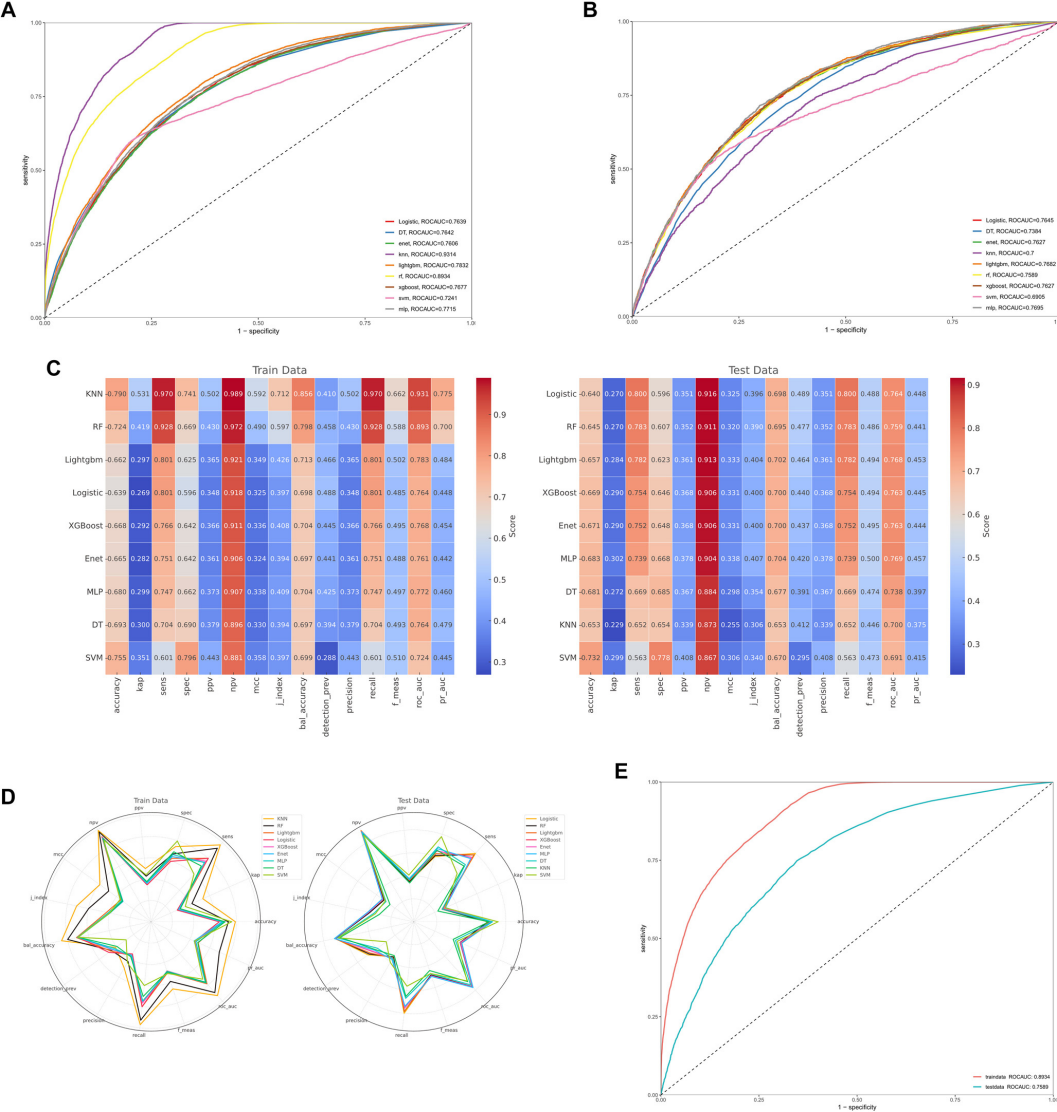
**Evaluation and comparison of machine learning models.** (A and B) ROC curves for nine machine learning models (RF, LightGBM, DT, XGBoost, MLP, SVM, KNN, ENET, Logistic) in both the training set (A) and the test set (B); (C and D) Summarization model performance metrics; (E) ROC curves for highlighting the superior predictive performance of the RF model. RF: Random forest; LightGBM: Light Gradient Boosting Machine; DT: Decision Tree; XGBoost: Extreme Gradient Boosting; MLP: Multilayer perceptron; SVM: Support vector machine; KNN: k-Nearest Neighbors; ENET: Elastic Net; ROC: Receiver operating characteristic.

### Subgroup analysis

Subgroup analysis based on Model 3 evaluated the consistency of associations between CBC-derived biomarkers and OAB across various demographic and clinical strata, including gender, age, race, education level, marital status, PIR, BMI, smoking status, alcohol use, hypertension, hyperlipidemia, diabetes, and cardiovascular disease. Forest plots showed a consistently positive association between elevated CBC-derived inflammatory biomarkers (SII, NHR, and MLR) and OAB risk across all subgroups (e.g., gender, age, race, education level; *P*-interaction > 0.05; Figure 3). Notably, hyperlipidemia was the only factor that significantly modified the relationship between specific biomarkers (SIRI, NLR, NMLR) and OAB risk (*P*-interaction < 0.05), while no significant interaction effects were observed for the other stratification variables.

### Boruta and LASSO regression

A total of 35,394 participants were divided into a training set of 24,775 and a testing set of 10,619, following a 7:3 ratio to support robust model development and validation. Feature selection was conducted using two complementary methods: the Boruta algorithm, an extension of RF, and LASSO regression. Boruta, a wrapper method around RF that iteratively evaluates feature importance, identified 19 key predictors: NMLR, MLR, NLR, NHR, SIRI, SII, CHD, diabetes, hyperlipidemia, hypertension, alcohol use, smoking status, BMI, PIR, marital status, education level, race, age, and gender (Figure 4A). Meanwhile, LASSO regression—a penalized method that performs variable selection by applying an L1 penalty to regression coefficients—identified 12 significant features: gender, age, race, education, marital status, PIR, BMI, smoking status, hypertension, diabetes, SII, and SIRI (Figure 4B and 4C). A comparative analysis revealed a consensus set of 12 variables, which were subsequently used for model construction (Figure 4D).

### Model evaluation and comparison

Figure 5A shows the receiver operating characteristic (ROC) curves for nine machine learning models—RF, Light Gradient Boosting Machine (LightGBM), Decision Tree (DT), Extreme Gradient Boosting (XGBoost), Multilayer Perceptron (MLP), Support Vector Machine (SVM), k-Nearest Neighbors (KNN), Elastic Net (ENET), and Logistic Regression—evaluated on the training set. Figure 5B presents the corresponding ROC curves for these models on the independent test set. Performance benchmarking indicated that RF and KNN exhibited the strongest predictive capabilities. RF achieved area under the curve (AUC) values of 0.89 on the training set and 0.76 on the test set, while KNN reached 0.93 and 0.70, respectively. Comprehensive performance metrics—including accuracy, sensitivity, positive predictive value, and negative predictive value—were compared between the training and test cohorts, as summarized in Figure 5C and 5D. Among all models, RF consistently outperformed the others across both datasets. Its ROC curve was separately highlighted in Figure 5E to emphasize its robust predictive ability, underscoring its effectiveness in capturing complex relationships between CBC-derived biomarkers and OAB risk.

## Discussion

The current study provides compelling evidence that systemic inflammation, as quantified by readily accessible CBC-derived biomarkers, is closely associated with the risk of OAB [23]. Our comprehensive analysis of 15 years of NHANES data demonstrates that elevated levels of SII, SIRI, NLR, MLR, and NMLR are significantly linked to OAB, even after adjusting for multiple confounders [24]. These findings suggest that chronic, low-grade inflammation may play a key role in OAB pathogenesis—a hypothesis further supported by the nonlinear relationships observed between biomarker levels and OAB incidence. Importantly, our results align with and extend prior histopathological evidence of localized bladder inflammation in OAB [25, 26]. Previous studies have reported mast cell infiltration, elevated pro-inflammatory cytokines (e.g., IL-6, TNF-α), and neuronal sensitization in bladder biopsies from both neurogenic and non-neurogenic OAB patients [27, 28]. We propose that systemic inflammation—reflected by increased neutrophil, monocyte, and platelet activity—may contribute to or exacerbate this local inflammatory milieu. Circulating mediators such as IL-1β or CRP could permeate the bladder mucosa, activating resident immune cells and amplifying tissue-level inflammation, thus linking systemic immune dysregulation to bladder hypersensitivity [29]. The weaker association observed for NHR (OR ═ 1.11, *P* ═ 0.067) may reflect the dual role of HDL-C as both an anti-inflammatory mediator and a metabolic regulator. HDL-C’s ability to suppress cytokine signaling and neutralize oxidative stress could mitigate neutrophil-driven inflammation, especially in individuals with metabolic comorbidities such as hyperlipidemia [30–32]. Additionally, unmeasured variability in HDL functionality or the use of lipid-lowering therapies may account for the attenuated effect size. Future studies incorporating lipid subfractions and longitudinal biomarker profiles are needed to better clarify NHR’s role in OAB pathophysiology. To validate these associations and identify the most predictive biomarkers, we employed advanced machine learning techniques, including an RF model and the extended Boruta algorithm. These methods identified SII and SIRI as the most robust predictors of OAB risk (AUC ═ 0.89 in training, 0.76 in testing), reinforcing their potential clinical utility in identifying inflammation-driven bladder dysfunction [33]. Collectively, our findings not only validate the role of inflammation in OAB but also highlight the potential of CBC-derived biomarkers as noninvasive, cost-effective screening tools in clinical settings. The biological plausibility of our findings is supported by the established roles of neutrophils, monocytes, and lymphocytes in inflammatory responses. Neutrophils are central to acute inflammation and tissue repair, while monocytes contribute to chronic inflammation by differentiating into macrophages and releasing pro-inflammatory cytokines [34, 35]. Lymphocytes, crucial for adaptive immunity, also help regulate inflammatory processes [36]. An imbalance in these cell types—captured by ratios, such as NLR and MLR—may reflect a proinflammatory state that predisposes individuals to tissue dysfunction, including altered bladder sensory signaling. This inflammatory environment can sensitize peripheral nerves, leading to the urgency and frequency characteristic of OAB. The nonlinear associations revealed in our threshold effect analysis suggest the presence of critical inflection points at which inflammation becomes pathological [37]. This pattern mirrors that seen in other chronic conditions, where a tipping point in systemic inflammation triggers clinical symptoms. Thus, our data provide a plausible mechanistic link between subclinical systemic inflammation and the development of OAB, underscoring the need for further research into the molecular pathways involved.

Methodologically, our study presents several strengths that enhance the credibility of its findings. The use of the NHANES database—with its nationally representative sample and rigorous data collection protocols—ensures broad generalizability to the U.S. population. Additionally, the large sample size and comprehensive statistical adjustments for a wide range of confounders—including age, gender, race, socioeconomic status, and comorbid conditions—minimize potential bias. The integration of both conventional logistic regression and advanced machine learning techniques (e.g., RF, LASSO, and Boruta) further strengthens the robustness of our results [38]. These methods enhance variable selection and improve predictive accuracy, as demonstrated by the high area under the ROC curve achieved by the RF model [39]. In combining traditional epidemiologic approaches with modern data science tools, our study offers a model for addressing complex clinical questions and advancing big data research into multifactorial disease mechanisms. Despite these strengths, several limitations warrant consideration. Most notably, the cross-sectional design of NHANES precludes causal inference. While we observed a strong association between elevated inflammatory biomarkers and OAB, it remains uncertain whether inflammation is a cause or consequence of bladder dysfunction. Residual confounding, despite our comprehensive covariate adjustment, may still influence the findings. Additionally, reliance on self-reported symptoms and questionnaire-based OAB diagnoses may introduce misclassification bias due to subjectivity in symptom perception and reporting. CBC-derived biomarkers, though practical indicators of systemic inflammation, are nonspecific and susceptible to influence by other conditions, such as infections, autoimmune disorders, or transient inflammatory states [40, 41]. Furthermore, while statistically significant thresholds were identified, these require validation in prospective cohorts and experimental studies to confirm clinical utility. The use of machine learning also introduces challenges—particularly the risk of overfitting and the necessity for external validation of model performance. These limitations underscore the need for longitudinal studies to clarify the temporal relationship between systemic inflammation and the onset of OAB. The clinical implications of our findings are substantial. Identifying CBC-derived inflammatory biomarkers as independent predictors of OAB risk creates opportunities for earlier detection and intervention. In practice, these biomarkers could be incorporated into standard laboratory panels, facilitating the identification of at-risk individuals before clinical symptoms emerge. This may allow for timely lifestyle modifications or targeted anti-inflammatory treatments, potentially slowing OAB progression and improving outcomes. Furthermore, machine learning-based predictive models could support individualized risk assessments and help tailor treatment strategies [42, 43]. Given the significant economic and quality-of-life burdens associated with OAB, such strategies could yield considerable public health benefits. Future research should examine the efficacy of anti-inflammatory interventions among individuals with elevated CBC-derived markers and determine whether reducing systemic inflammation translates to meaningful improvements in OAB symptoms and healthcare utilization. In conclusion, our study provides compelling evidence supporting the role of systemic inflammation in the pathogenesis of OAB. By integrating large-scale epidemiological data with cutting-edge statistical and machine learning methods, we identified nuanced dose-response relationships between inflammatory markers and OAB risk. These findings deepen our understanding of OAB pathophysiology and lay the groundwork for innovative diagnostic and therapeutic approaches. Moving forward, we plan to implement systematic model tuning and validation using independent datasets in prospective cohort studies. Interventional trials targeting systemic inflammation may ultimately reveal whether modulating the inflammatory response constitutes an effective treatment strategy for OAB.

## Conclusion

This study highlights a significant association between systemic inflammation—measured by CBC-derived biomarkers—and the risk of OAB. Elevated levels of markers, such as SII and SIRI were strongly linked to OAB, suggesting a pivotal role for inflammation in its pathogenesis. The application of machine learning models reinforced the predictive value of these biomarkers, supporting their potential use in early OAB detection. While these findings are promising, further longitudinal studies are needed to establish causality. Ultimately, integrating these biomarkers into clinical practice could enable earlier intervention and improve management strategies for patients with OAB.

## Supplemental data

**Table S1:** Standardized operationalization of NHANES symptom frequency. Metrics into OABSS

**NHANES symptom frequency metrics**	**OABSS**
Urgency urinary incontinence frequency	Urgency urinary incontinence score
Never	0
Less than once a month	1
A few times a month	1
A few times a night	2
Every day or night	3
Nocturia frequency	Nocturia score
0	0
1	1
2	2
3	3
4	3
5 or more	3

OABSS: Overactive bladder symptom score; NHANES: National Health and Nutrition Examination Survey.

**Table S2:** Sensitivity analysis of CBC-derived biomarkers under MNAR assumptions

**Biomarker**	**Original analysis (OR, 95% CI)**	**MNAR (+0.5 SD) (OR, 95% CI)**	**MNAR (−0.5 SD) (OR, 95% CI)**	**Δ% (original analysis vs MNAR)**
SII	1.21 (1.10–1.34)	1.18 (1.06–1.31)	1.25 (1.13–1.38)	−2.5% / +3.3%
SIRI	1.28 (1.16–1.41)	1.22 (1.10–1.35)	1.34 (1.21–1.48)	−4.7% / +4.7%
NLR	1.30 (1.18–1.44)	1.26 (1.14–1.39)	1.33 (1.20–1.47)	−3.1% / +2.3%
MLR	1.27 (1.15–1.40)	1.21 (1.09–1.34)	1.31 (1.19–1.44)	−4.7% / +3.1%
NMLR	1.31 (1.19–1.44)	1.25 (1.13–1.38)	1.36 (1.23–1.50)	−4.6% / +3.8%

MNAR: Missing-not-at-random; SII: Systemic Immune-Inflammation Index; SIRI: Systemic Inflammation Response Index; NHR: Neutrophil-to-High-Density Lipoprotein Ratio; NLR: Neutrophil-to-Lymphocyte Ratio; MLR: Monocyte-to-Lymphocyte Ratio; NMLR: Neutrophil-MLR.

## Data Availability

The datasets used in this study are publicly accessible through the National Health and Nutrition Examination Survey (NHANES) database, available at: https://www.cdc.gov/nchs/nhanes/index.htm.

## References

[1] Drake MJ (2018). Fundamentals of terminology in lower urinary tract function. Neurourol Urodyn.

[2] Mostafaei H, Janisch F, Mori K, Quhal F, Pradere B, Hajebrahimi S (2022). Placebo response in patients with oral therapy for overactive bladder: a systematic review and meta-analysis. Eur Urol Focus.

[3] Wang Y, Xu K, Hu H, Zhang X, Wang X, Na Y (2011). Prevalence, risk factors, and impact on health related quality of life of overactive bladder in China. Neurourol Urodyn.

[4] Stewart WF, Van Rooyen JB, Cundiff GW, Abrams P, Herzog AR, Corey R (2003). Prevalence and burden of overactive bladder in the United States. World J Urol.

[5] Raju R, Linder BJ (2020). Evaluation and treatment of overactive bladder in women. Mayo Clin Proc.

[6] Reynolds WS, Fowke J, Dmochowski R (2016). The burden of overactive bladder on US public health. Curr Bladder Dysfunct Rep.

[7] Durden E, Walker D, Gray S, Fowler R, Juneau P, Gooch K (2018). The economic burden of overactive bladder (OAB) and its effects on the costs associated with other chronic, age-related comorbidities in the United States. Neurourol Urodyn.

[8] Wei B, Zhao Y, Lin P, Qiu W, Wang S, Gu C (2024). The association between overactive bladder and systemic immunity-inflammation index: a cross-sectional study of NHANES 2005 to 2018. Sci Rep.

[9] Peyronnet B, Mironska E, Chapple C, Cardozo L, Oelke M, Dmochowski R (2019). A comprehensive review of overactive bladder pathophysiology: on the way to tailored treatment. Eur Urol.

[10] Wang CC, Jiang YH, Kuo HC (2020). The pharmacological mechanism of diabetes mellitus-associated overactive bladder and its treatment with botulinum toxin A. Toxins.

[11] He Q, Wu L, Deng C, He J, Wen J, Wei C (2024). Diabetes mellitus, systemic inflammation and overactive bladder. Front Endocrinol.

[12] Lemmon B, Kyrgiou M, Mullins E, Khullar V (2024). Cytokines in bladder pain syndrome: a review of the literature. Int Urogynecol J.

[13] King DE, Carek P, Mainous AG III, Pearson WS (2003). Inflammatory markers and exercise: differences related to exercise type. Med Sci Sports Exerc.

[14] Loprinzi PD, Ramulu PY (2013). Objectively measured physical activity and inflammatory markers among US adults with diabetes: implications for attenuating disease progression. Mayo Clin Proc.

[15] Zhou F, Wu L, Shen G, Chen X, Liu C, Huang D (2024). Association between monocyte to high-density lipoprotein-cholesterol ratio and osteoporosis: an analysis of the national health and nutrition examination survey 2013-2014. J Investig Med.

[16] Cao R, Li C, Geng F, Pan Y (2024). J-shaped association between systemic immune-inflammation index and periodontitis: results from NHANES 2009-2014. J Periodontol.

[17] Shi C, Cao H, Zeng G, Yang L, Wang Y (2024). The relationship between complete blood cell count-derived inflammatory biomarkers and benign prostatic hyperplasia in middle-aged and elderly individuals in the United States: evidence from NHANES 2001-2008. PloS One.

[18] Zuo R, Zhu F, Zhang C, Ma J, Chen J, Yue P (2023). The response prediction and prognostic values of systemic inflammation response index in patients with advanced lung adenocarcinoma. Thoracic Cancer.

[19] Tang L, Deng Y, Lai J, Guo X, Liu P, Li S (2023). Predictive effect of system inflammation response index for progression of chronic kidney disease in non-dialyzing patient. J Inflam Res.

[20] Blaivas JG, Panagopoulos G, Weiss JP, Somaroo C (2007). Validation of the overactive bladder symptom score. J Urol.

[21] Zhu S, Wang Z, Tao Z, Wang S, Wang Z (2023). Relationship between marijuana use and overactive bladder (OAB): a cross-sectional research of NHANES 2005 to 2018. Amer J Med.

[22] Muntner P, Hardy ST, Fine LJ, Jaeger BC, Wozniak G, Levitan EB (2020). Trends in blood pressure control among US adults with hypertension, 1999-2000 to 2017-2018. JAMA.

[23] Shoji F, Kozuma Y, Toyokawa G, Yamazaki K, Takeo S (2020). Complete blood cell count-derived inflammatory biomarkers in early-stage non-small-cell lung cancer. Ann Thorac Cardiovasc Surg.

[24] Yang X, Yin H, Xiao C, Li R, Liu Y (2022). The prognostic significance of C-reactive protein to albumin ratio in patients with severe fever with thrombocytopenia syndrome. Front Med.

[25] Jhang JF, Hsu YH, Jiang YH, Ho HC, Kuo HC (2021). Clinical relevance of bladder histopathological findings and their impact on treatment outcomes among patients with interstitial cystitis/bladder pain syndrome: an investigation of the European Society for the Study of Interstitial Cystitis histopathological classification. J Urol.

[26] Chang YC, Yu CY, Dong C, Chen SL, Sung WW (2024). Divergent histopathological and molecular patterns in chemically induced interstitial cystitis/bladder pain syndrome rat models. Sci Rep.

[27] Jiang YH, Jhang JF, Hsu YH, Kuo HC (2022). Usefulness of urinary biomarkers for assessing bladder condition and histopathology in patients with interstitial cystitis/bladder pain syndrome. Int J Mol Sci.

[28] Chen MC, Keshavan P, Gregory GD, Klumpp DJ (2007). RANTES mediates TNF-dependent lamina propria mast cell accumulation and barrier dysfunction in neurogenic cystitis. Amer J Physiol Renal Physiol.

[29] Jiang YH, Peng CH, Liu HT, Kuo HC (2013). Increased pro-inflammatory cytokines, C-reactive protein and nerve growth factor expressions in serum of patients with interstitial cystitis/bladder pain syndrome. PLoS One.

[30] Sohrabi Y, Schwarz D, Reinecke H (2022). LDL-C augments whereas HDL-C prevents inflammatory innate immune memory. Trends Mol Med.

[31] Varol E, Bas HA, Aksoy F, Ari H, Ozaydin M (2014). Relationship between neutrophil-lymphocyte ratio and isolated low high-density lipoprotein cholesterol. Angiology.

[32] Nicoară DM, Munteanu AI, Scutca AC, Mang N, Juganaru I, Brad GF (2023). Assessing the relationship between systemic immune-inflammation index and metabolic syndrome in children with obesity. Int J Mol Sci.

[33] Liao J, Wang L, Duan L, Gong F, Zhu H, Pan H (2025). Association between estimated glucose disposal rate and cardiovascular diseases in patients with diabetes or prediabetes: a cross-sectional study. Cardiovasc Diabetol.

[34] Zhang Y, Han S, Duan Z, Tian X, Li X, Hou G (2024). Associations of systemic inflammation and systemic immune inflammation with serum uric acid concentration and hyperuricemia risk: the mediating effect of body mass index. Front Endocrinol.

[35] Cheng CK, Chan J, Cembrowski GS, van Assendelft OW (2004). Complete blood count reference interval diagrams derived from NHANES III: stratification by age, sex, and race. Lab Hematol.

[36] Franchin M, Muscato P, Piffaretti G, Tozzi M (2024). Systemic inflammation index as useful tool to predict arteriovenous graft stenosis: our experience and literature review. J Vasc Access.

[37] Zahedi H, Atayie F, Samii Kondrud F, Balali A, Beyene J, Tahery N (2024). Associations of abdominal obesity with different types of bone fractures in adults: a systematic review and dose-response meta-analysis of prospective cohort studies. Crit Rev Food Sci Nutr.

[38] Gong Y, Ding W, Wang P, Wu Q, Yao X, Yang Q (2023). Evaluating machine learning methods of analyzing multiclass metabolomics. J Chem Inf Model.

[39] Kokla M, Virtanen J, Kolehmainen M, Paananen J, Hanhineva K (2019). Random forest-based imputation outperforms other methods for imputing LC-MS metabolomics data: a comparative study. BMC Bioinformatics.

[40] Zhang Y, Li T, Chen Q, Shen M, Fu X, Liu C (2024). The relationship between complete blood cell count-derived inflammatory biomarkers and erectile dysfunction in the United States. Sci Rep.

[41] Ke J, Qiu F, Fan W, Wei S (2023). Associations of complete blood cell count-derived inflammatory biomarkers with asthma and mortality in adults: a population-based study. Front Immunol.

[42] Ogunyemi O, Kermah D.

[43] Pekkala T, Hall A, Lötjönen J, Mattila J, Soininen H, Ngandu T (2017). Development of a late-life dementia prediction index with supervised machine learning in the population-based CAIDE study. J Alzheimer’s Dis.

